# A Simple Working Classification for Effective Surgical Management of Prominent Ears

**DOI:** 10.7759/cureus.77740

**Published:** 2025-01-20

**Authors:** Ramya Sree P, Rajat Gupta, Priya Bansal, Gautam Chaudhury

**Affiliations:** 1 Plastic Surgery, Excel Hospital, New Delhi, IND

**Keywords:** antihelix formation, conchal setback, otoplasty, prominent ears, scaphoconchal sutures

## Abstract

Prominent ear or prominauris is the most common congenital deformity of the ear. Children may get bullied in school because of this, and patients usually seek treatment for prominent ears because of psychosocial concerns even though functionally there are no issues. Numerous surgical procedures are described for correction of prominauris but there is no single procedure that gives consistent and satisfying outcomes. There are different classifications proposed but there is no simple classification described that would guide surgeons to better identify the deformity and to make a decision on specific surgical steps to be followed accordingly to give satisfactory and consistent results. We propose a classification to grade prominauris into three grades which will help in the decision-making in the surgical steps required for its correction.

## Introduction

The term prominent ears, otherwise known as prominauris, or bat ears, refers to ears that, regardless of size, “stick out” enough to appear abnormal. Prominent ears, being the most common congenital abnormality of the external ear, are of autosomal dominant inheritance with variable penetrance [1].

Patients seek correction of the deformity as they perceive it to be causing psychosocial disturbance, although there are no functional problems involved [2]. Patients with prominent ears present with a widened conchomastoid angle, often accompanied by the absence or underdevelopment of the antihelix [3]. These are two key characteristics that typically contribute to the prominence of the ear. The conchomastoid angle, which refers to the angle between the ear’s concha and the mastoid, tends to be more open in patients with prominent ears, while the antihelix, the inner fold of cartilage that helps shape the ear, may be underdeveloped or absent altogether. These anatomical features are often the primary concerns in patients with moderate to mild ear deformities [4].

Determining the extent of the deformity is the most crucial step in the surgical planning process. The literature suggests a number of classifications depending on the site involved [3]. The correction of prominent ears involves different surgical steps depending on the severity of the deformity [5]. There isn't a classification that would help surgeons in choosing the surgical techniques that would result in better and more consistent outcomes. We have attempted to develop a simpler and effective classification according to the severity of the condition.

## Technical report

Anatomical considerations

**Figure 1:**
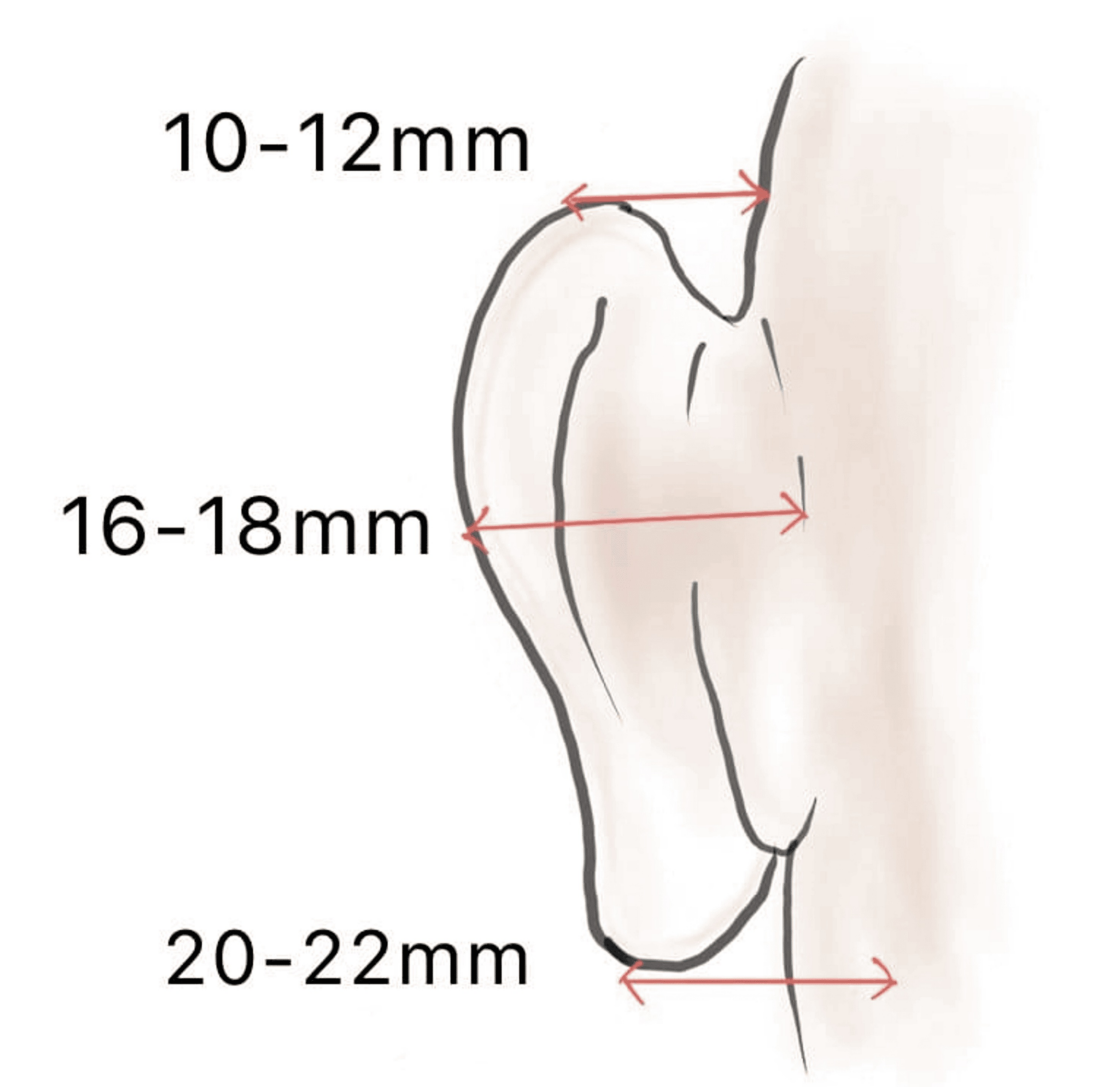
Normal projection of antihelix from the mastoid Image credits: Authors

In the normal ear, the concha makes a 90-degree angle with the head, resulting in ear projection. The cartilage folds 90 degrees backward at the scaphoconchal junction, creating the antihelix and bringing the ear parallel to the back of the head [3]. Changes in the shape of the auricle begin with the formation of the helix, followed by the antihelix and superior and inferior crura by six months of intrauterine life. Lack of development of the antihelical fold is the most common cause of the prominent ear [1]. The ear develops rapidly immediately after birth and reaches 90% of the adult size by one year of age and 99% by the age of 10 years. The normal projection of the helix from the mastoid is 10-12 mm at the superior helix, 16-18 mm at the midhelix, and 20-22 mm at the cauda helicis (Figure 1). The patients in whom there is a distance of more than 2 cm between the mastoid and the helix were found to have cartilage abnormalities [6].

Classification

Deformities accounting for the prominent ear are increased conchomastoid angle, underdeveloped antihelix fold, and a big conchal bowl. In our clinical experience, we have observed that the majority of patients seeking surgical correction for prominent ear deformities present with a widened concho-mastoid angle, often accompanied by the absence or underdevelopment of the antihelix. These anatomical features are often the primary concerns in patients with moderate to mild ear deformities. However, patients can present with a more severe deformity. In the instance of one patient, the deformity was so pronounced that it required the excision of part of the concha.

It's essential to identify the type of deformity when examining the patient. To assist plastic surgeons in identifying issues and organizing surgical steps to correct deformities, we suggest a grading system for prominent ears (Table 1). With this approach, we grade the severity according to the patient's presenting deformity.

This classification can now help in decision-making in the surgical steps required for the correction of prominent ears. There are three grades and each can be managed in the following way: (i) Grade 1 deformity: Conchal set back with conchomastoid sutures as described by Furnas [4]; (ii) Grade 2 deformity: Anti-helical fold creation along with conchal set back. We prefer the suturing technique as described by Mustarde [7]; Grade 3 deformity: Excision of conchal cartilage along with the above procedures. A grade 3 deformity typically involves excessive conchal prominence, which can only be corrected through surgical resection of the conchal cartilage.

**Table 1:** Working classification of prominent ear deformity

Grade	Deformity	Surgical steps required
Grade 1	Increased concho-mastoid angle	Chonchal setback
Grade 2	Increased concho-mastoid angle	Chonchal setback
	Absent antihelical fold	Formation of antihelix
Grade 3	Increased concho-mastoid angle	Chonchal setback
	Absent antihelical fold	Formation of antihelix
	Deep conchal bowl	Conchal excision

Correction of prominent earlobe

In most patients, correction of the prominent earlobe is mandatory along with the above procedures for better patient satisfaction. The procedure is done under local or general anesthesia depending on the age of the patient. Preoperatively, the skin to be excised and the anticipated site of the antihelical fold are marked.

Skin Excision

**Figure 2:**
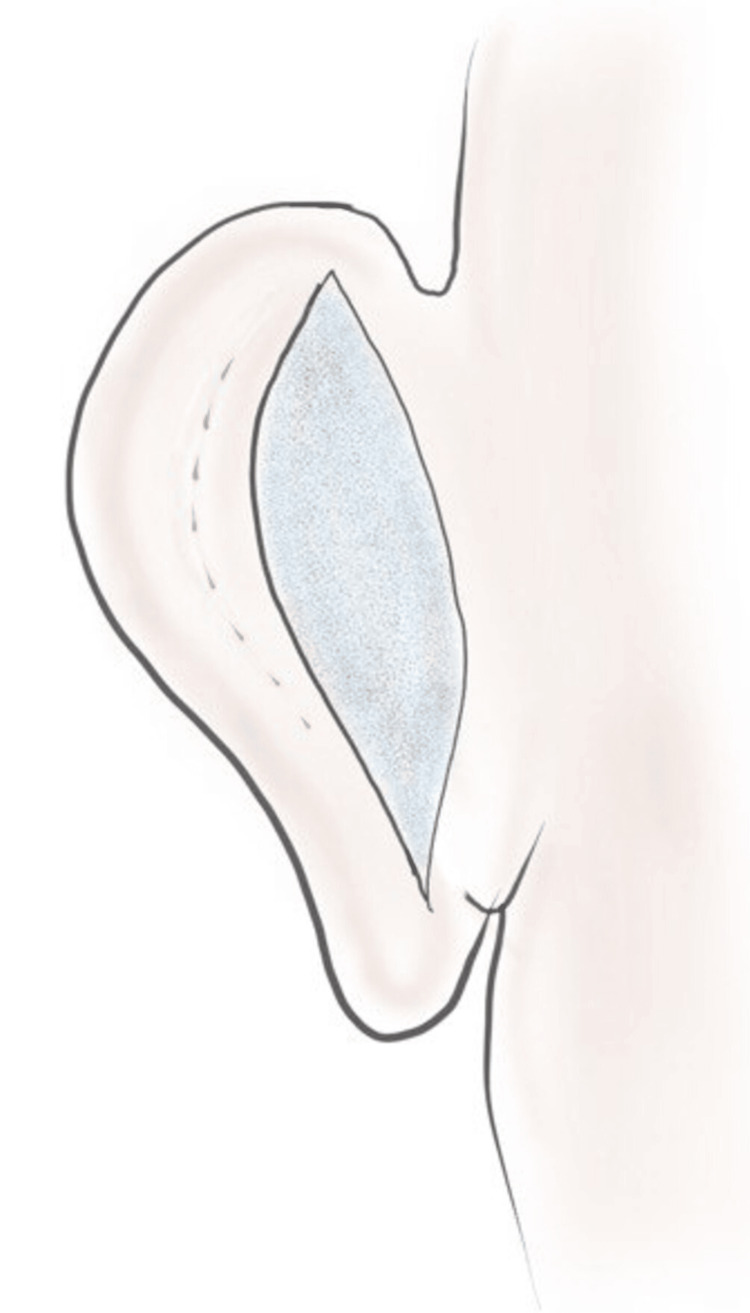
Skin excision over the posterior aspect; proposed site of antihelix is marked Image credits: Authors

An elliptical incision is made over the posterior aspect of the concha according to the preoperative markings (Figure 2). The skin is excised, and the perichondrium is elevated to expose the conchal cartilage. The skin flap over the mastoid region is undermined, and the postauricular muscle is divided to expose the mastoid fascia.

Formation of Antihelix

**Figure 3:**
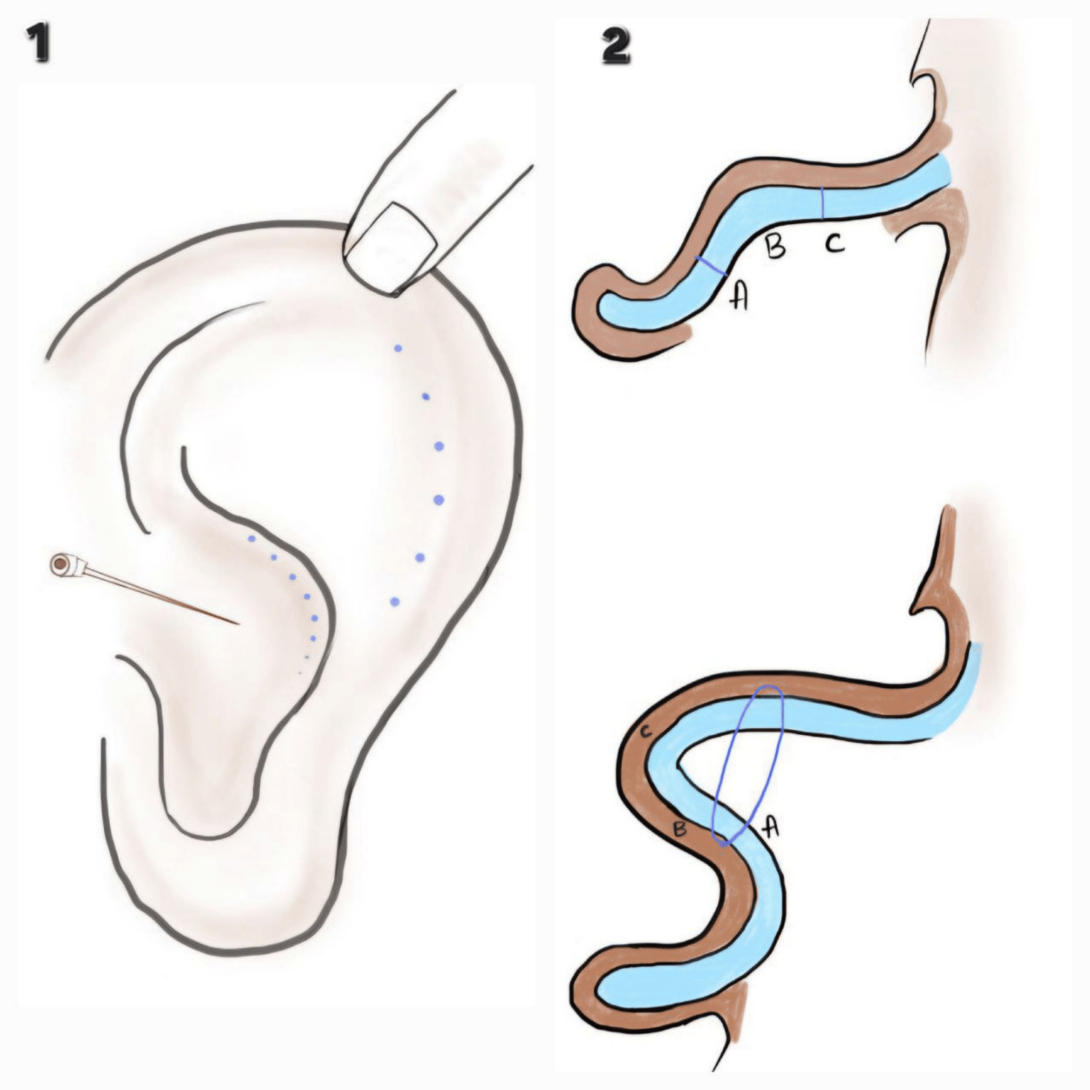
Formation of antihelix. 1. Marking of the antihelix; 2: Suturing of the cartilage for antihelix formation Image credits: Authors

The antihelix is created using Mustarde’s technique. The helix is pushed back using a finger to form the desired antihelix projection. The planned suture points are marked by tattooing on the cartilage by passing the methylene blue dipped needle through and through (Figure 3). Using 4-0 polypropylene, three to four horizontal mattress sutures are placed across the designated locations, avoiding the anterior skin, and are knotted one after the other while preserving the antihelix projection.

Conchal Excision

**Figure 4:**
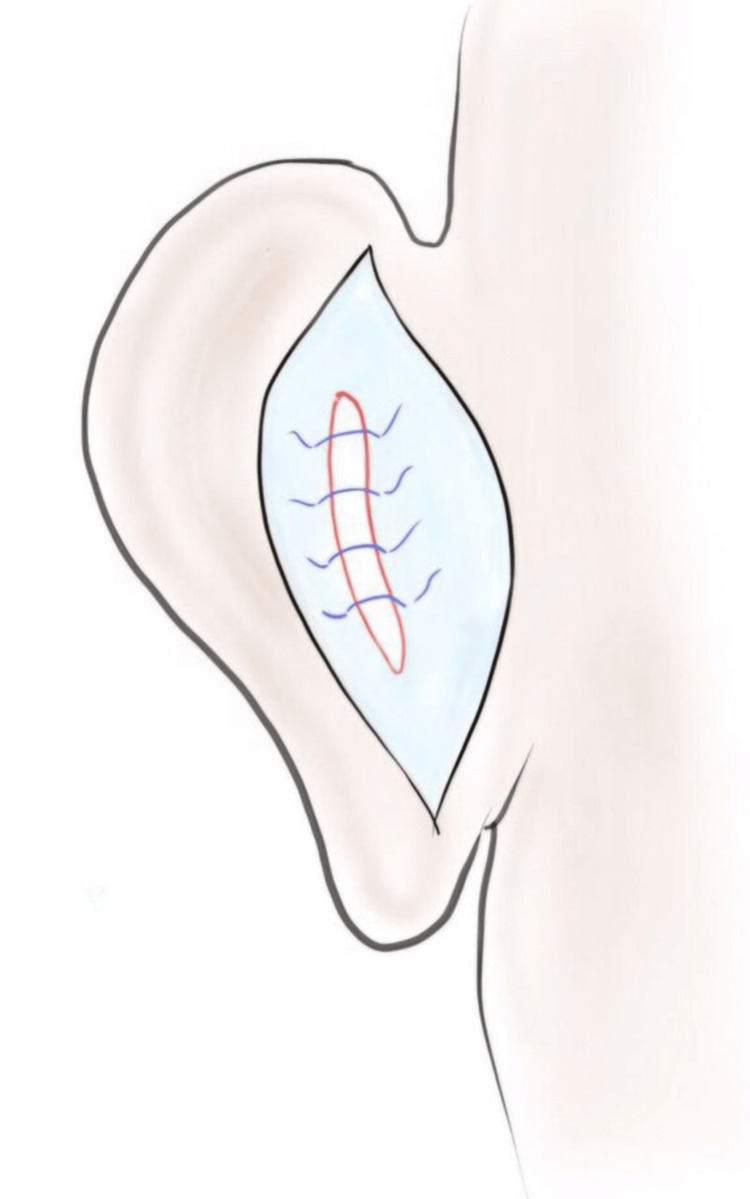
Excision of the excesss cartilage in the conchal bowl and suturing of the cartilage ends. Image credits: Authors

In patients with a deep conchal bowl, a kidney-shaped cartilage is excised preserving the anterior skin. The cut ends of the cartilage were sutured using absorbable sutures (Figure 4). Depending on the excess cartilage, the width of the excision is determined intraoperatively.

Conchal Setback

**Figure 5:**
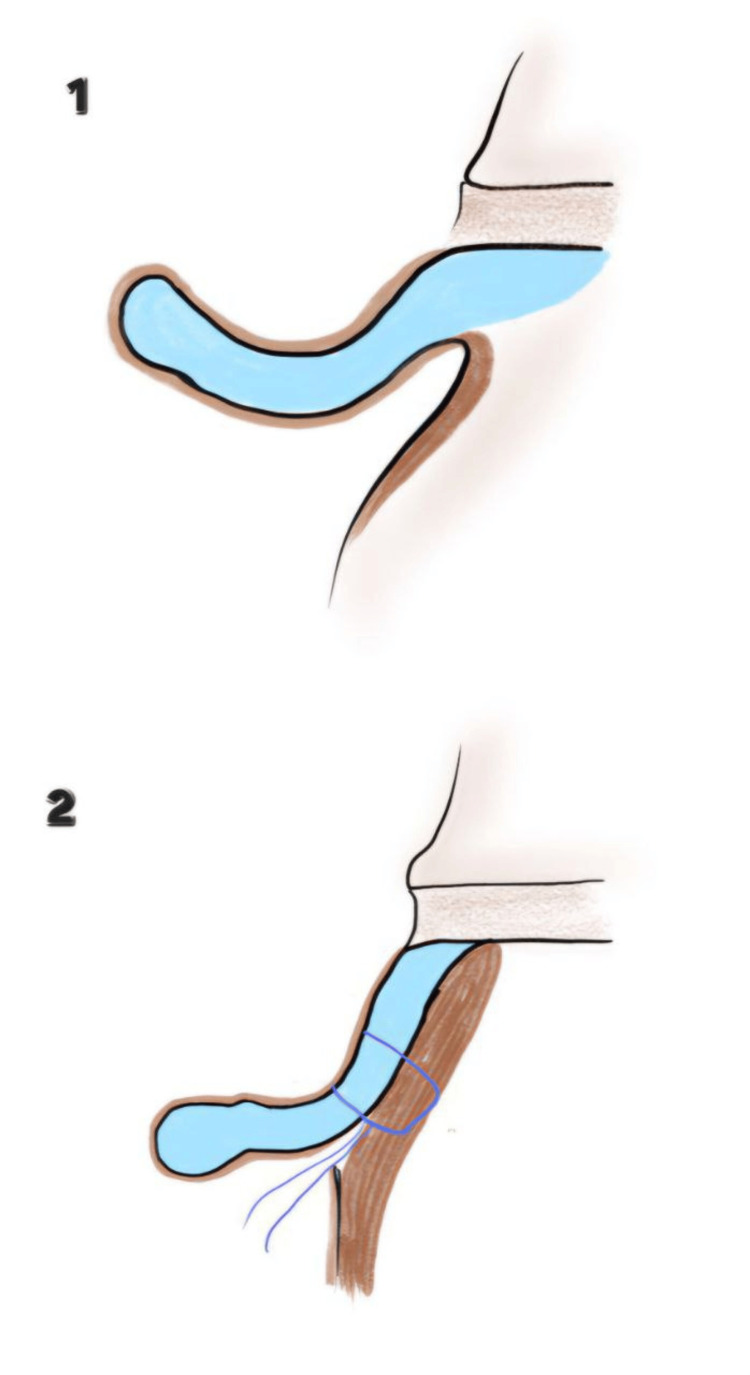
(1) projection of the ear in prominent ear; (2) Projection of the ear after conchomastoid sutures Image credits: Authors

The conchal setback is done using the Frunas technique. Two to three conchomastoid sutures are planned to obliterate the space between the concha and the skull; 2-0 polydioxone sutures are placed from the conchal cartilage to the mastoid fascia at the proposed sites and are sequentially knotted (Figure 5). Care must be taken while placing the sutures over the mastoid fascia as improper placement of the sutures might constrict the auditory canal. Since a mild loosening of the sutures was observed over the course of three to four months, a slight overcorrection is performed.

Complications

The early complications of otoplasty include hematoma and infection, which might lead to pressure necrosis of the underlying cartilage, wound dehiscence, and skin necrosis. Late complications include scarring, asymmetry, suture extrusion, and aesthetic complications [8].

Our experience

**Figure 6:**
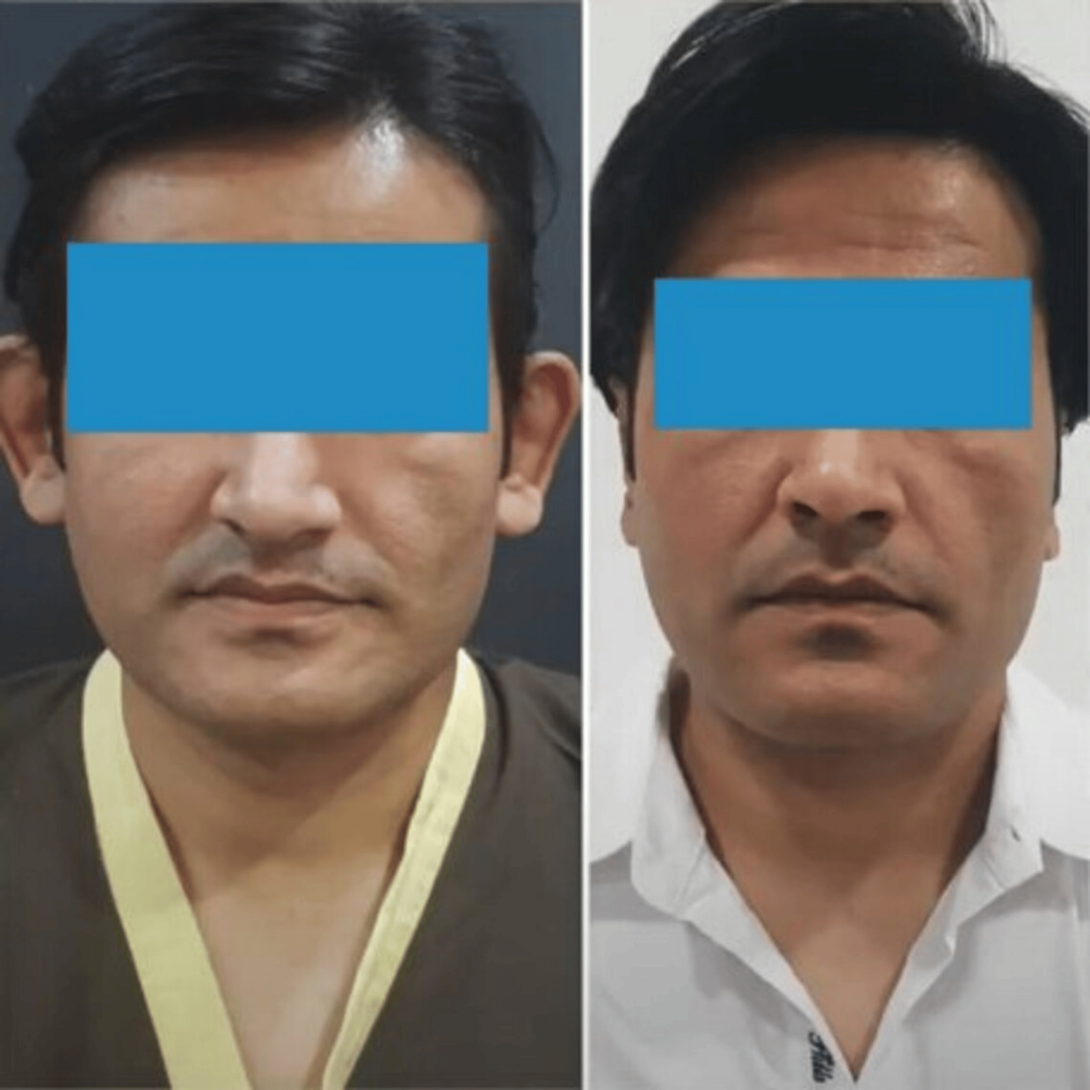
Preoperative and postoperative picture in the anterior view (Patient 1)

**Figure 7:**
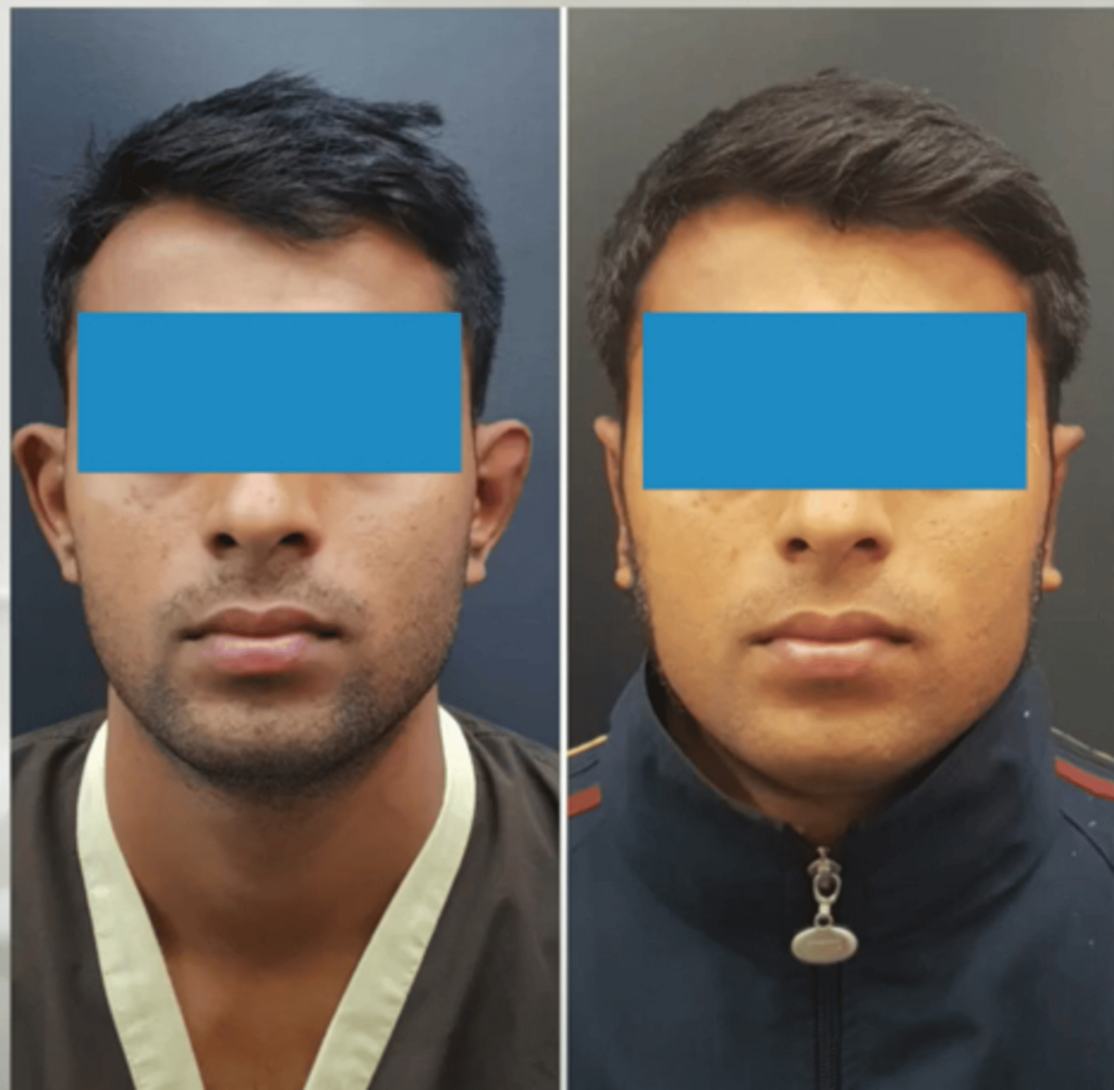
Preoperative and postoperative picture in the anterior view (Patient 2)

**Figure 8:**
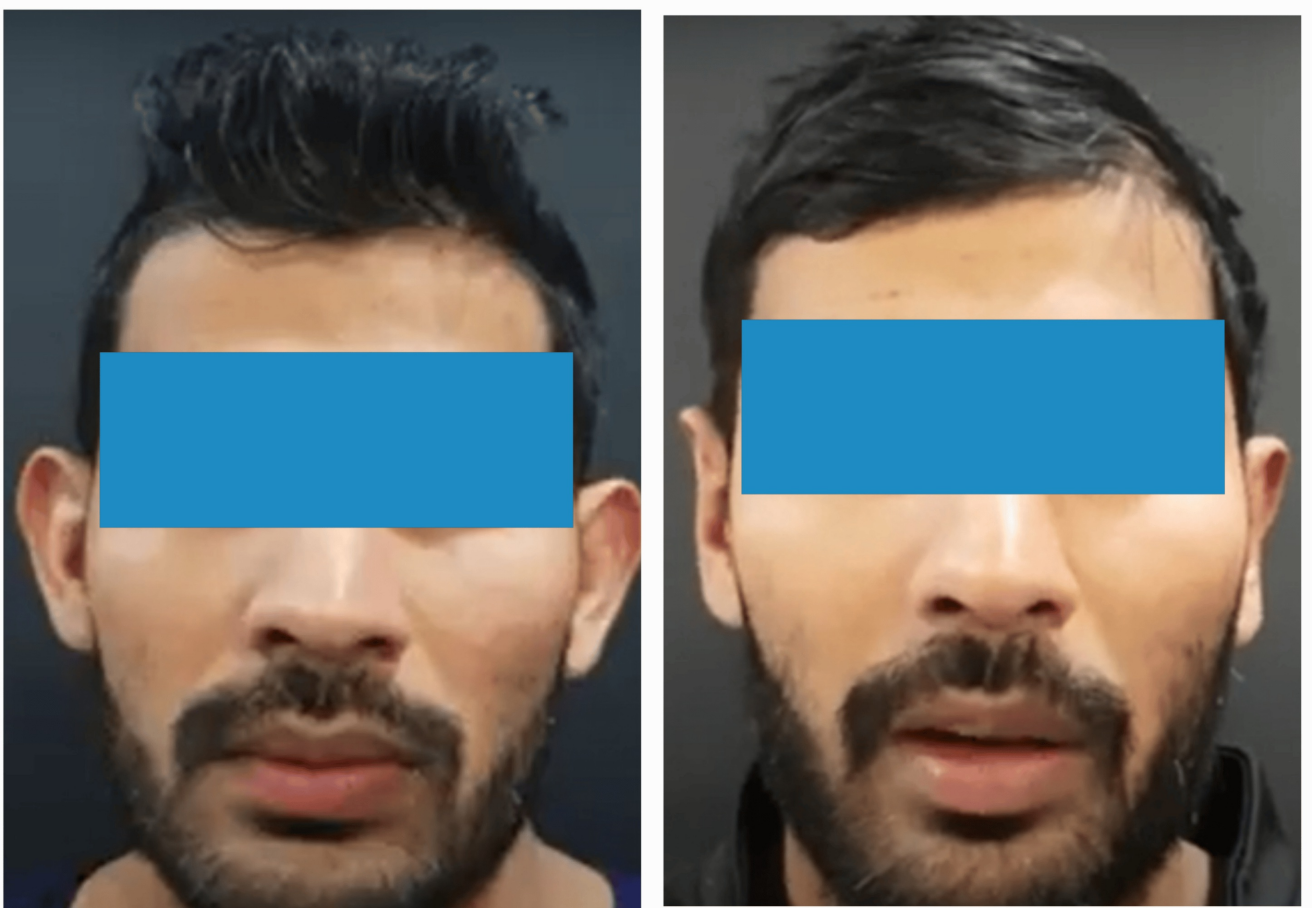
Preoperative and postoperative picture in the anterior view (Patient 3)

In the last three years, we have operated on 72 patients who had prominent ears. A total of 29 patients in grade 1 underwent conchal setback surgery, 42 patients in grade 2 underwent antihelical fold formation along with conchal setback surgery, and one patient in grade 3 had conchal excision. All our patients had satisfactory outcomes and are happy with the results. Three cases are shown in Figures 6-8.

One patient of grade 1 had a recurrence due to loosening of the conchomastoid sutures six months postoperatively. The patient is pleased with the outcome of the conchal setback that was performed at six months. Suture extrusion was noticed in seven patients after a period of six to seven months postoperatively. Extruded sutures were removed without deforming the contour. There were no instances of hematomas, infection, or skin or cartilage necrosis.

## Discussion

The auricular cartilage is composed of elastic cartilage which gives definition and support to the skin and soft tissues of the ear. It is a three-dimensional structure with soft curvatures that has many elevations, depressions, and folds in a specific manner [8]. Hence understanding the deformity is important for choosing a particular procedure. Numerous procedures were described in the literature for the same but no single procedure can give consistent and aesthetically pleasing results [9].

Ear reconstruction for mutilated ears was described in 600 BC in Sushrutha Samhitha. Later in the 1800s, Ely used an anterior approach to do a full-thickness crescentic excision. Using a posterior technique, Morestein excised skin and underlying cartilage while preserving the anterior skin [10]. In one instance, he secured the ear in place with non-absorbable sutures. Mustarde later refined this procedure in 1963 by positioning the nonabsorbable sutures posteriorly without interfering with the cartilage to create the antihelical fold [6]. The method of suturing the perichondrium to the mastoid fascia was first described by Gersuny in 1903 and subsequently refined by Frunas in 1968 [10]. He used the technique where the entire thickness of the auricular cartilage is sutured to the mastoid periosteum [5]. This reduces the excessive cupping of the conchal bowl by closing the gap between the concha and the mastoid prominence.

The lack of an antihelix in the prominent ear was initially noted by Luckett, who then tried to rectify it by excision of cartilage from the suggested antihelix position and securing it with mattress sutures. The cartilage excision created a sharper antihelix and a more unnatural appearance [10]. Becker recommended posteriorly placing mattress sutures and cutting a transverse portion of the cartilage [1]. The concept of interlocking stress in cartilage was explained by Gibson and Davis, especially subperichondrial scoring on one surface would allow the cartilage to warp in the opposite way [11]. Based on this, numerous sectional and scoring procedures were devised.

The available techniques for the correction of prominent ears are grouped as cartilage sparing, sculpting, combination techniques, flap procedures, and lobule correction techniques [12,13]. We prefer the cartilage-sparing technique for antihelical fold creation using Mustarde’s technique [6].

Despite the existing research, only a limited number of papers have suggested a simple grading system for the deformity [3], which would facilitate an easier understanding of the condition. The identification of the deformity aids in the appropriate planning of the procedure and produces a surgical result that is both aesthetically pleasing and predictable. In this report, we suggested a simple grading system that will assist plastic surgeons in accurately planning the required corrections.

Patients with Grade 1 deformities in our system will just require a conchal setback. Patients in grade 2 will need a conchal setback procedure as well as anti-helical fold creation. In addition to conchal setback and antihelical fold formation, grade 3 patients will need excess conchal excision. The majority of patients require correction of the earlobe in addition to other procedures.

Limitations

This working classification has been used in our institute with consistent outcomes. However, as of now, it is a single-institute experience. Other surgeons from different institutes can use this classification to know the consistency of the results in a larger sample population.

## Conclusions

To achieve optimal and aesthetically pleasing results in ear surgery, a multi-step surgical approach is often necessary. The exact combination of procedures varies based on the specific characteristics and severity of the ear deformity in each patient. Surgical correction may include steps such as reducing the concho-mastoid angle, reshaping the antihelix, or excising part of the concha, depending on the individual’s needs.

The decision-making process in choosing which surgical steps to perform is guided by the severity of the deformity and the goals of achieving the most natural-looking ear shape. To aid in this decision-making, we have developed a classification system. This classification serves as a valuable tool for plastic surgeons, allowing them to assess the deformity more accurately and select the appropriate surgical techniques. By following this framework, surgeons can consistently achieve satisfying, predictable outcomes for patients undergoing ear deformity correction.
